# Emotional Contexts Exert a Distracting Effect on Attention and Inhibitory Control in Female and Male Adolescents

**DOI:** 10.1038/s41598-017-02020-8

**Published:** 2017-05-18

**Authors:** Julieta Ramos-Loyo, Luis A. Llamas-Alonso, Andrés A. González-Garrido, Juan Hernández-Villalobos

**Affiliations:** Instituto de Neurociencias, Universidad de Guadalajara, Guadalajara, Jalisco Mexico

## Abstract

Adolescents exhibit difficulties in behavioral regulation that become more evident when emotional contexts are involved, since these may hinder the development of socially-adaptive behaviors. The objectives of the present study were: to examine the influence of emotional contexts on adolescents’ ability to inhibit a prepotent response, evaluated by ERPs, and to determine whether sex differences in response inhibition are observed in adolescents in those contexts. Participants performed a prepotent response inhibition task (Go-NoGo) under 3 background context conditions: neutral, pleasant, and unpleasant. While no differences in accuracy were observed, the presence of emotional contexts did prolong reaction times compared to the neutral context. Also, the unpleasant context caused an enhancement of N2 amplitudes compared to the neutral and pleasant contexts. Also, N2 and P3 latencies were longer in emotional contexts than in the neutral condition during both correct responses and correct inhibitions. No sex differences were found in amplitude, but females showed longer N2 and P3 latencies than males. These results confirm the idea that, in adolescents, unpleasant pictures receive preferential attention over neutral images and so generate greater difficulty in response inhibition. Finally, results demonstrate that sex differences in inhibition control in adolescence were observed only in relation to time-processing.

## Introduction

Adolescence is a developmental period in which many physical, cognitive, emotional and physiological changes take place. It is widely-known that this transitional period between childhood and adulthood is often characterized by behavioral and emotional problems that can lead young people into risky situations and make them highly vulnerable to psychiatric illnesses and substance dependence^1^. Difficulties in behavioral regulation in this population sector may become even more apparent when a romantic partner or peers are present, or when individuals are involved in emotionally-charged events. One biological factor that contributes to these difficulties is the incomplete maturation of the prefrontal lobes, together with over-activation of subcortical-limbic regions associated with affective responses and gratification^2–9^. This disequilibrium in the developmental processes of the limbic system and the prefrontal cortex underlies an imbalance between two systems: affective bottom-up regulation, and top-down impulse control^10^.

According to Barkley^11^, behavioral regulation depends on the executive functions, but the inhibition processes that constitute a core component of those functions have not yet fully matured during adolescence. In fact, lower activity in the neural structures involved in response inhibition –including the right inferior frontal gyrus, the left dorsal and medial frontal areas and the cingulate gyrus, among others– has been described as being predictive of future involvement in such problem behaviors as consuming alcohol and other substances of abuse^12^.

Managing daily life situations, meanwhile, demands that individuals perceive multiple environmental stimuli, and entails exerting attentional control and inhibiting prepotent responses in order to produce socially-adaptive behavior. Emotional stimuli in particular have a natural saliency that attracts attention and so modulates inhibitory control. One important factor to take into account in studies that seek to explore the effects of valence on cognition is the level of arousal that pictures with emotional content produce in an individual, as this could modulate the interference effect on the accuracy of both response and response inhibition^13^.

Some neuroimaging studies have addressed the effects of emotional stimuli on response inhibition in relation to brain activation. Two main interacting neural circuits are thought to be involved in emotional inhibition processing. The first is engaged-in-response inhibition, which involves the frontoparietal network; the other is a cortico-limbic circuit related to the top-down regulation of emotional stimuli^14–16^. In this regard, Brown *et al*.’s^17^ study with adults found higher activation of the inferior frontal gyrus (IFC) during inhibition trials compared to targets, and even higher levels when unpleasant pictures –instead of neutral ones– were presented as the background. Using a similar approach, Brown *et al*.^18^ subsequently conducted a study with adolescents. In the latter case, they found no differences in error inhibition rates, but determined that reaction times were shorter in neutral contexts than unpleasant ones. Their fMRI data revealed that the activation patterns of adolescents differed from those of adults, as the DLPFC and regions of the VLPFC showed greater activation in neutral than unpleasant contexts. These data suggest that differences between adolescents and adults may reflect not only contrasts in the activation level of those areas, but differences in certain neural structures involved in inhibition processing in the presence of emotional contexts.

The Go-NoGo paradigm is one of the most widely-used approaches to evaluate inhibition processing. Its aim is to generate a prepotent response by presenting a high rate of target stimuli (Go) to which the subject should respond, and a low rate of rare stimuli (NoGo) from which subjects should withhold their response^19^. The recording of brain electrical activity temporally-synchronized with specific stimuli (ERP) makes it possible to analyze precise temporal changes in cognitive and emotional processing. For this reason, the ERP technique has been used to examine the effects of emotional stimuli on inhibition processes. In this regard, two main ERP components have been identified in relation to response inhibition: N2 and P3. N2 occurs between 200 and 400 ms after stimulus onset and shows a maximum in frontal regions. The N2Go component is interpreted as reflecting responses to novelty and attentional control, whereas the N2NoGo would be more closely related to conflict resolution and inhibition processes associated with the functioning of the frontal and anterior cingulate cortices^20–24^. Some studies have demonstrated that N2NoGo amplitudes rise when task difficulty increases by reducing the reaction time deadline (low difficulty = 1000 ms, medium = 500, and high = 300)^25^. Géczy, Czgler and Balázs^26^ proposed that increased N2 amplitudes in response to NoGo stimuli after Go cues could be related to more intense efforts to activate the response inhibition system and thus impede preparations for response execution. Another interesting assumption is that the N2NoGo reflects the closure of the inhibition process, or a response-monitoring process^27^. In summary, it is more likely that N2Go is associated with attentional control processes, while N2NoGo is related to cognitive control processes prior to response inhibition.

Turning to P3, this is a positive deflection which latency oscillates between 300 and 600 ms. It has been suggested that the parietal P3Go component is an analog of the target P3 or P3b component obtained in odd-ball paradigms^28^. Therefore, P3Go amplitude is enhanced at low probability targets, and has been related to such processes as stimulus evaluation, attention allocation, context updating, and subsequent memory storage^20, 27, 29, 30^. In contrast, the P3NoGo component emerges 300–600 ms after stimulus onset, but with a frontocentral distribution. P3NoGo amplitude decreases and its latency delays with rising task difficulty as working memory requirements increase^31^. Also, P3NoGo amplitude is higher for motor compared to non-motor inhibition tasks^32^. This component appears to be linked to the later stage of the inhibition process that is closely related to the inhibition of motor responses^20, 31–34^. It is generally accepted that the P3NoGo is related to the outcome of the inhibition process and reflects conflict inhibition processing,^35, 36^ or an evaluation of the inhibitory process itself^37^.

Few ERP studies have addressed the disruptive effect of emotional information on attentional control. In this regard, researchers have identified differences that are a function of emotional context valence; *i.e*., while larger P3 amplitudes were present in a positive context compared to a neutral one on NoGo trials, N2 showed higher responses in the negative context^38, 39^. Another study evaluated distinct effects of two negative emotional context valences (threat and blood stimuli) on a Go/No-Go task^40^. Those authors found that the NoGo-N2 was larger in response to the threat than to the blood stimuli, whereas NoGo-P3 amplitude did not differ between the two conditions. Their interpretation of these results was that the threat stimuli facilitated performance of a prepotent response and enhanced conflict monitoring when action must be withheld. Killgore, Oki and Yurgelun-Todd^41^, meanwhile, studied the effects of two different unpleasant negative contexts on response inhibition, observing that the difference waves of P3 amplitude under disgusting contexts were smaller than under fearful ones, suggesting that disgusting distracters consume more attentional resources and, therefore, impair inhibitory control to a greater extent than fearful ones. Data from the aforementioned studies suggest that implicit emotional contexts may distract attention and, in so doing, affect inhibition processes. However, the first studies cited^38–40^ were conducted with mixed groups of men and women and did not test for sex differences, while the latter work^42^ involved only women. It is our contention that studying and comparing female and male adolescents in relation to the effects that emotional contexts exert on response inhibition could reveal additional data on whether sex differences exist during this important period, when top-down control is maturing. Eventually, it may be possible to relate such differences to behavioral and emotional regulation in social environments.

On the topic of sex differences in adolescents, Shulman, Harden, Chein and Steinberg^43^ propose that the window of heightened vulnerability to risk-taking in this stage may be greater in magnitude and more protracted in males than females. This agrees with data from brain developmental studies that have revealed earlier brain development in females than males. Lenroot *et al*.^44^ observed that, for nearly all structures, the peak of gray matter volumes generally occurred approximately 1–2 years earlier in adolescent girls than adolescent boys, and that this difference was especially apparent in the frontal lobes. In addition, Killgore, Oki and Yurgelun-Todd^41^ found that at a higher age females show a progressive increase in left prefrontal cortex activation and a decrease in amygdala activation while processing fearful faces, but that males do not. This suggests a higher progressive modulation of the prefrontal cortex in the former compared to the latter. Unfortunately –and despite the importance of this topic– little attention has been paid to studying the effects of pleasant and unpleasant emotional contexts on inhibition processes in female and male adolescents, or to the neural mechanisms that underlie them.

We hypothesized that emotional contexts will have a deleterious effect on response inhibition processing, because their saliency draws attention and so will distract participants from the main task. Considering the higher modulation of the prefrontal cortex observed in female adolescents, it might be expected that they will show higher accuracy and lower neural activation than males during response inhibition when non-emotional stimuli are present. However, some studies have found that females are more sensitive than males to unpleasant/negative stimuli, while males are more reactive to pleasant/positive ones, especially images with erotic content^45–48^. Therefore, we could assume that unpleasant stimuli would cause greater inhibition interference in females, whereas males would find it more difficult to ignore pleasant stimuli while attempting to perform the inhibition task.

Based on this background, the objectives of the present study were twofold: (1) to examine the influence of emotional contexts on the ability to inhibit a prepotent response in adolescents, in relation to their behavioral performance and the time course of cognitive processing as measured by ERPs; and, (2) to elucidate whether sex differences in response inhibition under the emotional contexts are evident during adolescence.

## Results

### Behavioral results

#### Sex-related comparisons

Behavioral results are shown in Table 1. ANOVAs showed no between-sex differences in the behavioral data (percentage of correct responses: *F*(1,34) = 0.07, *p* = 0.79, *ŋ*_*p*_^*2*^ = 0.002; percentage of correct inhibitions: *F*(1,34) = 0.27, *p* = 0.60, *ŋ*_*p*_^*2*^ = 0.008; reaction times: *F*(1,34) = 2.40, *p* = 0.12, *ŋ*_*p*_^*2*^ = 0.06).

**Table 1:** Behavioral performance data in each condition: means and standard deviations.

	Correct responses (%)		Correct inhibitions (%)		Reaction times (ms)	
	Females	Males	Females	Males	Females	Males
Neutral	98.30 (4.30)	98.30 (3.10)	66.60 (12.20)	66.60 (11.70)	469.70 (46.10)	442.70 (65.50)
Pleasant	98.30 (2.50)	97.70 (4.80)	71.60 (9.50)	68.30 (12.30)	492.20 (58.90)	457.10 (61.20)
Unpleasant	97.20 (5.90)	98.30 (3.30)	70.00 (8.80)	63.30 (12.80)	484.40 (62.50)	453.60 (72.20)

#### Context-related performances

There were no statistically-significant differences in the percentage of correct responses (*F*(2,68) = 1.13, *p* = 0.32, *ŋ*_*p*_^*2*^ = 0.03) or correct inhibitions among conditions (*F*(2,68) = 1.20, *p* = 0.30, *ŋ*_*p*_^*2*^ = 0.03). However, the presence of emotional background contexts did prolong reaction times when compared to the neutral context (*F*(2,68) = 6.42, *p* = 0.003, *ŋ*_*p*_^*2*^ = 0.15). Behavioral data is presented in Table 1.

### ERP Results

#### Trial-related comparisons

**Figure 1:**
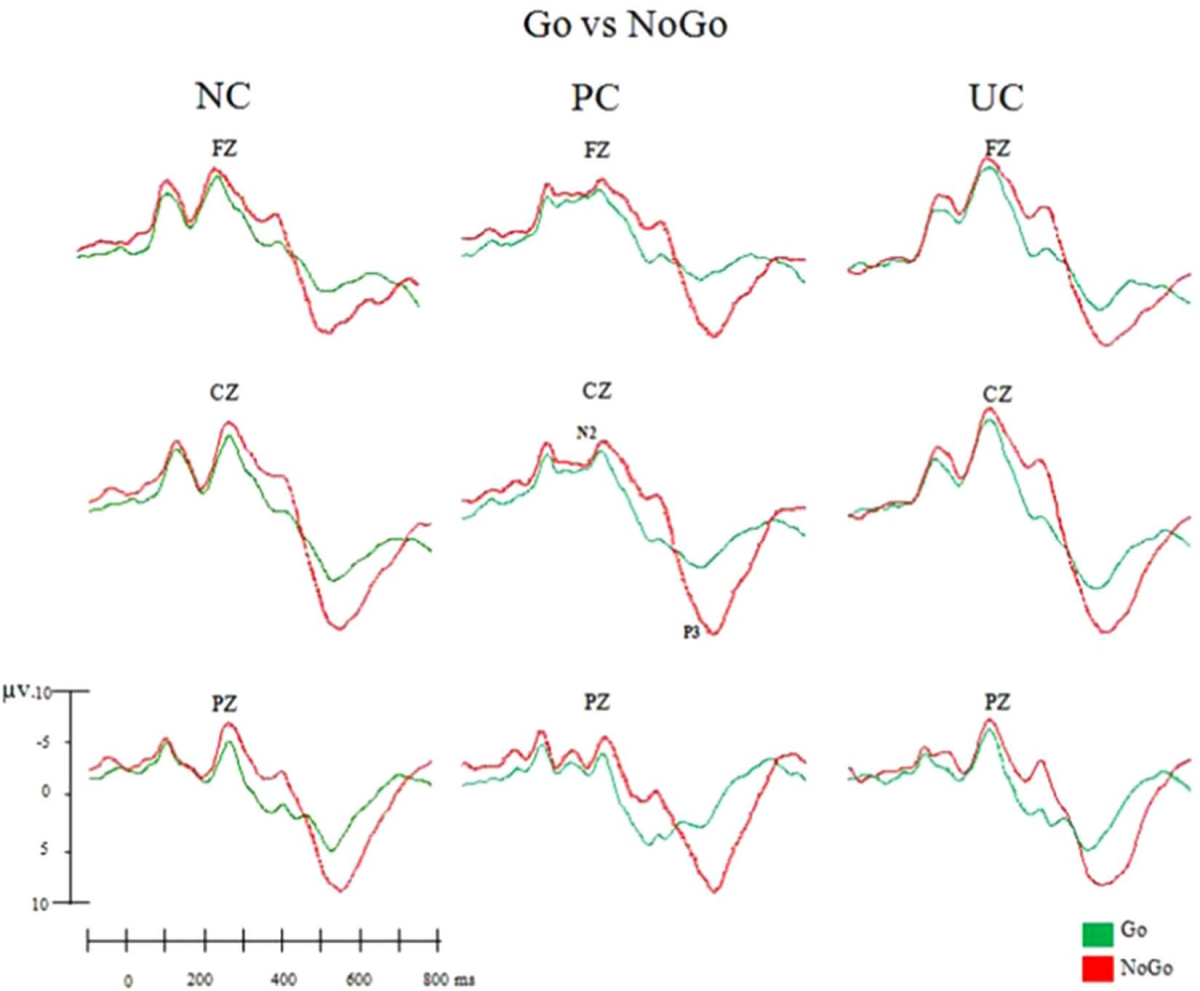
Comparison of ERP waveforms between Go and NoGo trials in each context condition: neutral (NC), pleasant (PC) and unpleasant (UC).

Grand-average ERP waveforms for the Go and NoGo trials in each condition are shown in Fig. 1. N2 and P3 amplitude and latency values in each condition for females and males are presented in Tables 2 and 3 in supplementary data.

#### Differences between Go/NoGo trials

***N2***: No significant differences were found with respect to the N2 amplitude (*F*(1,34) = 1.74, *p* = 0.19, *ŋ*_*p*_^*2*^ = 0.04) when comparing Go versus NoGo trials. However, N2 latency was longer during NoGo than Go trials (*F*(1,34) = 8.39, *p* = 0.007, *ŋ*_*p*_^*2*^ = 0.19).

***P3*** *:* The significant interactions found –(sex × trials × context: *F*(2,68) = 3.30, *p* = 0.04, *ŋ*_*p*_^*2*^ = 0.08; and sex × trials × topographic distribution: *F*(2,68) = 3.52, *p* = 0.03, *ŋ*_*p*_^*2*^ = 0.09)– show that both females and males had higher P3 amplitudes during NoGo than Go trials in all contexts (*p* < 0.001, in the three post hoc comparisons) and in all regions (post hoc *p* < 0.001, at frontal, central, and parietal leads).

The analysis of P3 latency showed two significant interactions: trials × context, *F*(2,68) = 4.26, *p* = 0.02, *ŋ*_*p*_^*2*^ = 0.11; and trials × topographic distribution, *F*(4,136) = 3.51, *p* = 0.04, *ŋ*_*p*_^*2*^ = 0.09. These results demonstrated prolonged P3 in NoGo trials in all contexts (*p* < 0.001 for all post hoc comparisons) and all the cerebral regions explored (*p* < 0.001 for post hoc comparisons in the frontal, central, and parietal areas).

#### Scalp distribution for the NoGo-ERPs

**N2NoGo:** The amplitude of this component showed significant differences with respect to its scalp distribution. A significant interaction of trials × topographic distribution (*F*(2,68) = 8.68, *p* < 0.001, *ŋ*_*p*_^*2*^ = 0.20) was found, and the post hoc analysis demonstrated that the N2NoGo amplitude was lower in the parietal than in the frontal (p < 0.001) and central areas (p = 0.002).

N2NoGo latency did not reach the level of significant differences regarding its topographic distribution (trials × topographic distribution, F(2,68) = 2.76, p = 0.07, ŋ_p_^2^ = 0.07).

**P3NoGo:** The analysis of the P3NoGo amplitude showed a significant interaction of sex × trials × topographic distribution (*F*(2,68) = 3.52, *p* = 0.03, *ŋ*_*p*_^*2*^ = 0.09). Post hoc comparisons revealed that P3NoGo showed higher voltages in the central areas than in the frontal and parietal regions (*p* = 0.008, in both comparisons); however, these differences were only observable in male participants. In addition, the post hoc analyses subsequent to the interaction trials × laterality (*F*(4,136) = 3.51, *p* = 0.04, *ŋ*_*p*_^*2*^ = 0.09) showed that P3NoGo had higher voltages at midline than in the left and right hemispheres in the central (*p* < 0.001, in both comparisons) and parietal regions (left: *p* = 0.002, right: *p* < 0.001).

When analyzing the P3NoGo latency, an interaction between trials and topographic distribution emerged (*F*(4,136) = 3.51, *p* = 0.04, *ŋ*_*p*_^*2*^ = 0.09). Post hoc comparisons demonstrated that P3NoGo latency was longer in the frontal than the central (*p* = 0.003) and parietal regions (*p* < 0.001).

#### Scalp distribution for the Go-ERPs

**N2Go:** The analysis of the amplitude of N2Go showed a significant interaction of trials × topographic distribution (*F*(2,68) = 8.68, *p* < 0.001, *ŋ*_*p*_^*2*^ = 0.20). Post hoc analyses suggested that N2Go amplitude was significantly lower in the parietal areas than in the frontal (*p* < 0.001) and central regions (*p* = 0.002).

The analysis of the N2Go latency did not reveal any significant differences between the conditions examined (trials × contexts interaction *F*(2,68) = 49, *p* = 0.54, *ŋ*_*p*_^*2*^ = 0.01).

**P3Go:** A significant interaction of sex × trials × topographic distribution (*F*(2,68) = 3.52, *p* = 0.03, *ŋ*_*p*_^*2*^ = 0.09) was found when analyzing the P3Go amplitude. Post hoc comparisons demonstrated that P3Go showed higher voltages in the frontal areas than in the central (*p* < 0.001), and parietal regions (*p* = 0.004) in both females and males. In addition, another significant interaction of trials × topographic distribution × laterality was found (*F*(4,136) = 41.86, *p* < 0.001, *ŋ*_*p*_^*2*^ = 0.55). Post hoc comparisons indicated that the P3Go voltage magnitude was higher at midline than in the right parietal area (*p* = 0.002).

The latency of the P3Go component also showed an interaction effect of trials × topographic distribution (*F*(4,136) = 3.51, *p* = 0.04, *ŋ*_*p*_^*2*^ = 0.09), which revealed that it was more prolonged in the frontal than the central (*p* = 0.003) and parietal regions (*p* < 0.001).

#### Differences among background contexts

**Figure 2:**
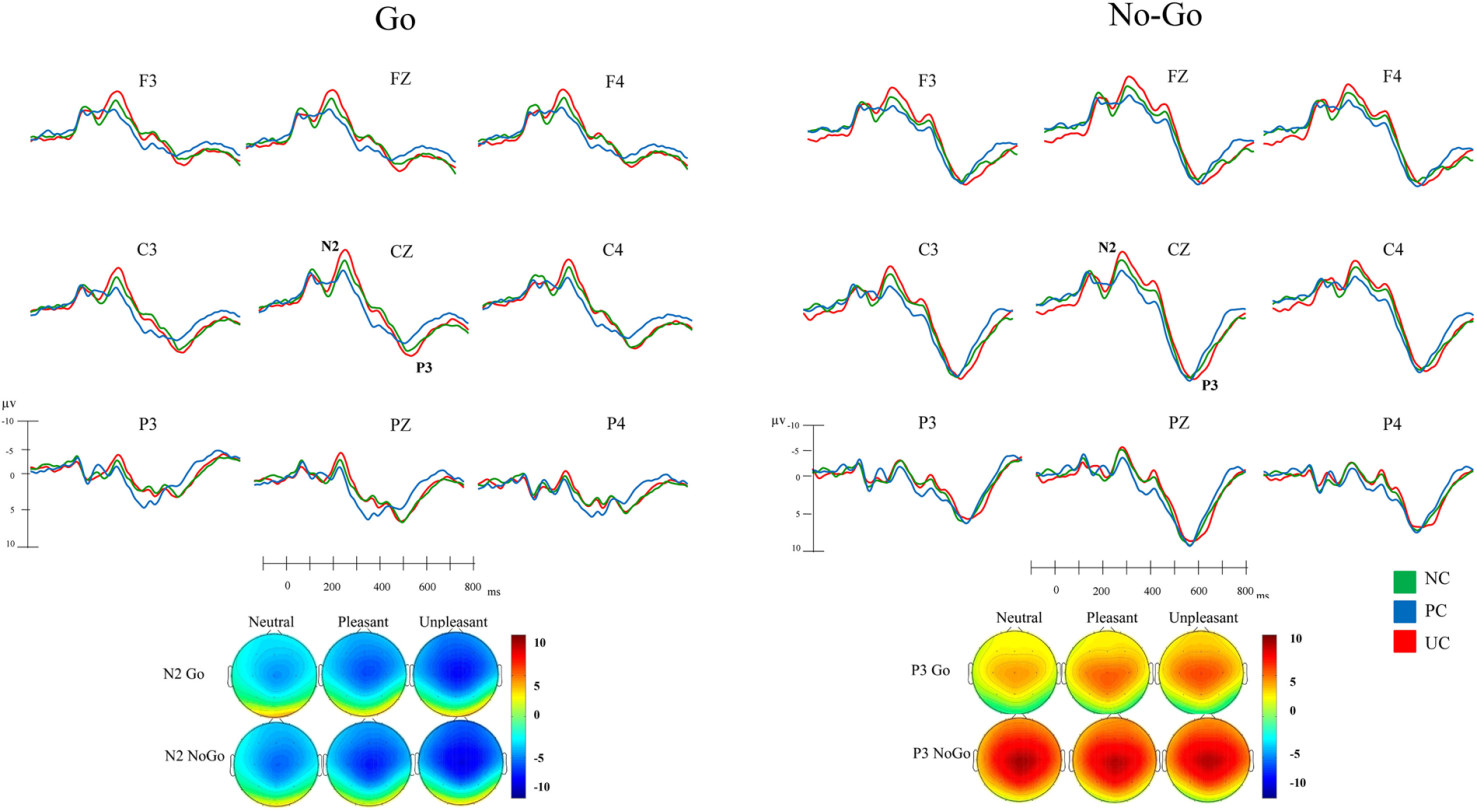
Top panel: ERP waveforms during Go and NoGo trials in each context condition: neutral (NC), pleasant (PC) and unpleasant (UC). Bottom panel: the topographical distribution of N2 and P3 is shown. The color scale represents microvolt values, with scales adjusted independently for N2 and P3. For N2, blue corresponds to maximum negativity; for P3, red corresponds to maximum positivity.

**N2**: An ANOVA performed with the data on N2 amplitudes showed a main effect of contexts (*F*(2,68) = 12.70, *p* < 0.001, *ŋ*_*p*_^*2*^ = 0.27). As it can be observed in Fig. 2, the unpleasant context had a higher amplitude than either the neutral (*p* = 0.001) or pleasant ones (*p* = 0.006). Also, a significant sex × topographic distribution × context interaction was found (*F*(4,136) = 2.93, *p* = 0.03, *ŋ*_*p*_^*2*^ = 0.08). Here, the post hoc analysis suggested that the differences between the contexts were restricted to females in the frontal and central areas (unpleasant > neutral, *p* = 0.001; unpleasant > pleasant, *p* = 0.004).

The analyses of the N2 latency demonstrated a significant effect of contexts (*F*(2,68) = 18.88, *p* < 0.001, *ŋ*_*p*_^*2*^ = 0.35), as latency was longer in the pleasant and unpleasant contexts than in the neutral one (*p* < 0.001, in both comparisons).

**P3**. The ANOVA performed for P3 amplitudes failed to demonstrate any significance among across contexts (*F*(2,68) = 2.26, *p* = 0.11, *ŋ*_*p*_^*2*^ = 0.06).

However, the P3 latency analysis was significant (*F*(2,68) = 15.34, *p* < 0.001, *ŋ*_*p*_^*2*^ = 0.31), as post hoc comparisons indicated that it was longer for both emotional contexts than for the neutral one (*p* < 0.001, in both comparisons).

#### Between-sex differences

**N2**. Regardless of trial type, the analysis of N2 amplitudes failed to find any significant sex-related differences (*F*(1,34) = 0.005, *p* = 0.94, *ŋ*_*p*_^*2*^ = 0.001). However, the same analysis for N2 latency demonstrated a laterality × sex interaction (*F*(2,68) = 3.48, *p* = 0.04, *ŋ*_*p*_^*2*^ = 0.09). Post hoc comparisons depicted a significantly longer latency for N2 in females than males that was spread widely across the scalp (left, *p* = 0.049; right, *p* = 0.009; midline, *p* < 0.001).

**P3**. Similarly, analyses of the P3 amplitude (*F*(1,34) = 0.06, *p* = 0.80, *ŋ*_*p*_^*2*^ = 0.002) revealed no significant sex-related differences.

In contrast, the analysis of the P3Go latency did show a significant sex × trials interaction (*F*(2,68) = 4.38, *p* = 0.02, *ŋ*_*p*_^*2*^ = 0.11), and subsequent post hoc comparisons suggested that females had longer P3Go latencies than males (*p* < 0.001).

## Discussion

The primary aim of the present study was to explore the effects that pleasant and unpleasant emotional contexts exert on the ability to inhibit a prepotent response in female and male adolescents in relation to behavioral performance and the time course of cognitive processing. Our data confirmed the results of previous studies in the sense that emotional pictures affect reaction times and ERP components during a response inhibition task. Sex differences were observed only in the ERP latencies.

No effect of context conditions was found in relation to behavioral accuracy, a result that might be explained as a top-down compensatory mechanism for the emotional interference. However, the presence of pleasant and unpleasant background contexts did prolong reaction times in relation to the neutral context condition. In this regard, Brown *et al*.^18^ also found longer reaction times in unpleasant contexts than neutral ones, Cohen-Gilbert and Thomas^49^ obtained shorter reaction times in positive *vs*. negative contexts and, Singhal *et al*.^50^ observed similar results with frightening *vs*. neutral distracters in adolescents. Although the adolescents in our study were able to maintain adequate attention levels (high rate of correct responses), when their results were compared to those of adults who had performed a similar task in a previous study^51^, they clearly showed lower inhibition abilities. While the adults obtained an overall (sex and condition) average of 84% for correct inhibitions, the adolescents reached only 64%. This poor inhibition accuracy was accompanied by faster responses in adolescents. These data suggest a higher susceptibility to interference from irrelevant stimuli in adolescents compared to adults; an effect that could be attributed to the incomplete maturation of the prefrontal cortex, together with enhanced activation of sub-cortical regions in the presence of affective stimuli^10, 52, 53^.

Turning to brain activity, the effects of emotional contexts observed in our work, agree with results from other studies in which emotional stimuli interfered with cognitive tasks^15, 38, 54, 55^. The unpleasant background context caused an enhancement of N2 amplitudes compared to the neutral and pleasant contexts, though P3 amplitude did not change as a function of context. In addition, N2 and P3 latencies were longer in both emotional contexts than in the neutral one. It is noteworthy that the context effects on ERPs were present during both correct responses (Go) and correct inhibitions (NoGo), with no differences between them regarding conditions. However, our results did replicate those described in the literature in relation to the higher P3NoGo amplitudes and longer latencies compared to the Go trials, a finding that has been interpreted as showing that inhibitory processes are more demanding than executive motor functions^56, 57^.

The increased N2 amplitudes in the unpleasant context may suggest that higher top-down attentional control was required to accomplish both response and response inhibition. These results concur with those found by other authors^39^. It has been reported that higher NoGoN2 amplitudes are associated with more efficient inhibitory control^51, 58^. In contrast, P3 amplitudes showed no significant differences among conditions in either the Go or NoGo trials. Albert *et al*.^39^ did observe larger P3 amplitudes in the positive context in NoGo trials and suggested that stopping responses to positive stimuli are more difficult than to negative ones. As well, Buodos *et al*.^40^ found that the NoGo-N2 was larger in response to the threatening stimuli than the blood stimuli, whereas NoGo-P3 amplitude did not differ between the two conditions. Their interpretation of these results was that the threatening stimuli enhanced conflict-monitoring related to response inhibition. However, while some authors have also associated P3NoGo with motor inhibition^59^, others indicate that it may be related to the evaluation of the inhibitory process^37, 60^. Therefore, it appears that these processes were less affected by the presence of emotional contexts in adolescents.

The longer N2 and P3 latencies observed for both emotional backgrounds compared to the neutral one indicate the need for additional processing time due to their saliency, which concurred with the longer reaction times observed in the emotional contexts. It has been reported that N2NoGo amplitude and latency also vary with task difficulty, since they increase as this parameter increases, while the NoGoP3 amplitude was not affected. In addition, the latencies of both components increased with the degree of task difficulty^60^. In the present study, the difficulty that adolescents experienced in achieving inhibition was precisely due to the need to inhibit, first, the interference from the background distracter and, second, from the prepotent response itself. This may be reflected in the increase in the N2 amplitude associated with attentional top-down control requirements. It may be that due to the saliency of the emotional stimuli, their distracter effect increased task difficulty, provoking the increase in N2 amplitude and the prolonged latencies of both components.

It is important to mention that while adults suffered a context effect only on the NoGo trials in a similar task, reflected in the enhancement of N2 amplitudes in the unpleasant compared to the neutral and pleasant contexts^51^, adolescents showed an overall effect in both Go and NoGoERPs. Therefore, the distracting effect of the emotional stimuli observed in adolescents indicates that they experienced difficulties in exercising interference control over the background emotional stimuli at different levels, including attentional control, conflict resolution and inhibition processes associated with the functioning of the frontal and anterior cingulate cortices^20–22^. The discrepancy in inhibition accuracy between adolescents and adults might be explained by an incomplete maturation of the top-down frontal mechanisms, which is even more visible when emotional backgrounds are present, suggesting that the activation of the limbic structures (bottom-up regulation) could add to adolescents’ difficulties with those mechanisms, as other authors have proposed^10^.

No significant effect of valence was observed in N2 amplitude. These data disagree with previous studies^61–63^, where a greater disrupting effect on attentional control of the unpleasant compared to the pleasant stimuli has been found. Those results support the assumption that an attentional bias toward negatively-valenced stimuli is due to their saliency for survival^64^. Alternatively, it is argued that the higher effect of negative stimuli depends on the level of arousal that emotional stimuli provoke in individual subjects^60^, which may explain the absence of valence differences, since in the present experiment the pleasant and unpleasant stimuli used were balanced in their levels of arousal.

On the other hand, no sex-based differences were found in the amplitudes of the brain responses of these adolescents. There is evidence that some areas of the brain, including the frontal lobes, develop earlier in girls than boys^44^. While this would predict better inhibition processing, there are also reports that contextual stimuli –especially negative ones– exert greater attraction in women than men^42, 48^. If this is true, then it could counteract women’s potentially better inhibition abilities. However, the data from the present study suggest that the deleterious effect of emotional contexts was similar in both sexes, though the female adolescents seemed to spend more time on stimuli processing, as shown by the differences in N2 and P3 latencies.

Since previous research on the effect of emotional contexts on inhibition processes was conducted using either ERPs with mixed groups of adults of both sexes^38–40^ or only with women^43^, or by means of fMRI that considered sex differences in adults^65^ or male adolescents^18^, it is not possible to directly compare our results with those of other studies. The most similar study is the previous one we performed in adults, since it involved a similar task and addressed sex differences^51^. Therefore, our results provide new clues to the effects of emotional stimuli on brain electrical activity during response and response inhibition when sex is considered as an important variable.

More research is needed to achieve a broader understanding of the brain mechanisms involved in the regulation of emotional stimuli in adolescents and adults, and of the factors that foster individual differences. Furthermore, studies considering adolescent groups with special conditions in which poor impulse control is a core characteristic –eg. impulsivity, ADHD, borderline personality, and substance abuse– would be very valuable in designing effective behavioral training programs and therapies designed to generate greater adaptive behavior in social environments.

## Conclusions

In summary, these results confirm the assumption that implicit, unpleasant pictures receive preferential attention over neutral images and so generate more difficulties in inhibition processing in adolescents. However, our findings only demonstrated sex differences related to the time processing of control inhibition in this population.

## Method

### Participants

A total of 36 right-handed teenagers participated in the study; 18 females (X = 16.89, SD = 0.48 years old) and 18 males (X = 16.88, SD = 0.65 years old). All were attending pre-college classes and showed above normal IQ according to the vocabulary and cubes WAIS sub-tests. The exclusion criteria were neurological or psychiatric antecedents, DSM-IV criteria for ADHD, and ongoing medical treatment. Though participation was voluntary, subjects did receive a modest financial compensation. All parents signed an informed consent form. The study was approved by the Ethical Board of the Institute of Neuroscience of the University of Guadalajara. All experiments in the present study were performed in accordance with approved guidelines and regulations.

### Stimuli and experimental procedures

**Figure 3:**
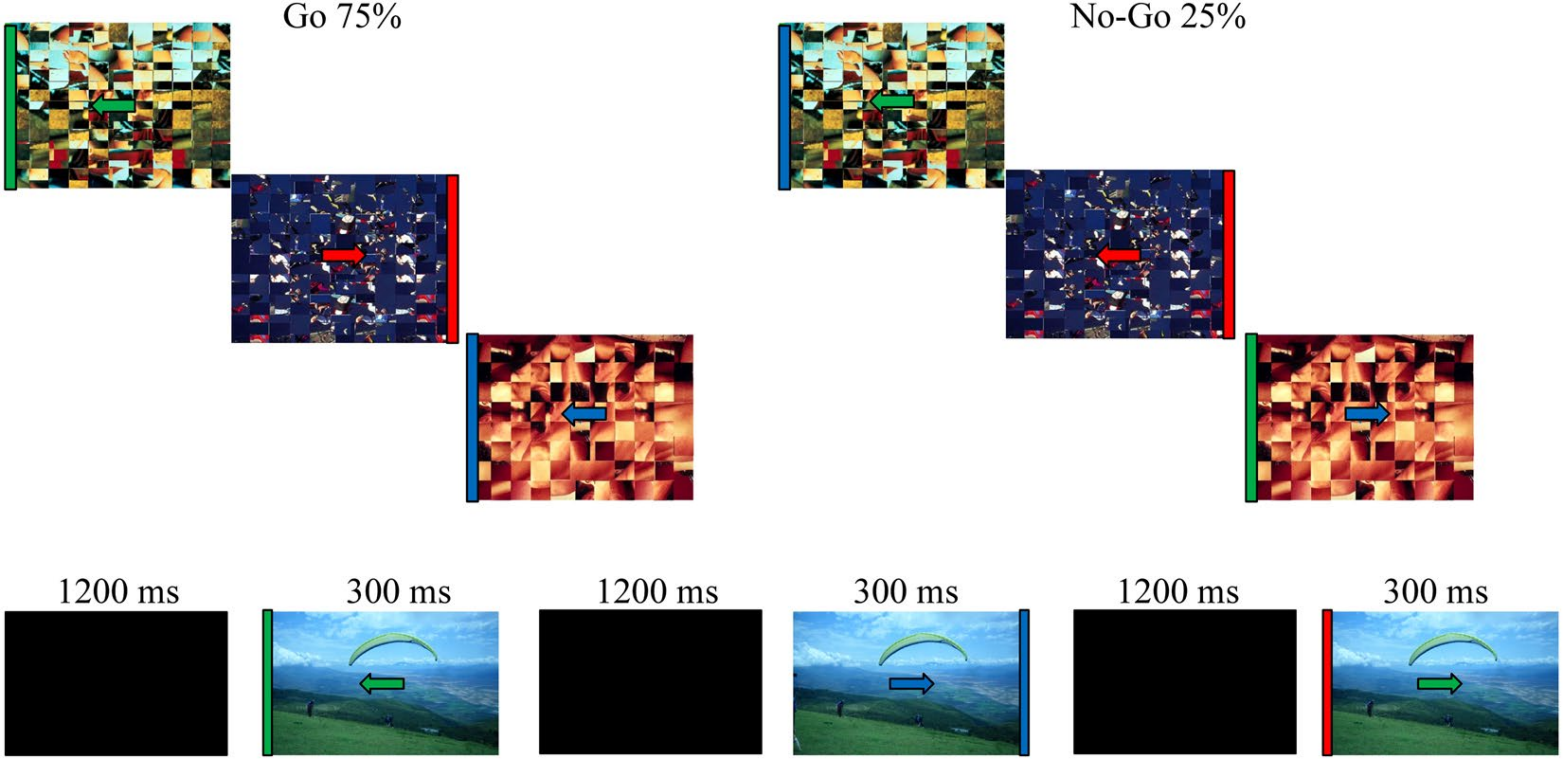
The Go/NoGo task paradigm. On the Go trials, the arrow and bar coincided in both direction and color, while on the NoGo trials they did not match. In the upper panel the arrow and bar are inserted in the neutral background context; in the lower panel the paradigm is represented with an example of a pleasant emotional stimulus.

Subjects were seated comfortably in a dimly-lit, sound-attenuated, electrically-shielded room. A Go/NoGo response inhibition task was performed under 3 context conditions: neutral, pleasant and unpleasant emotional contexts. The stimuli consisted of colored arrows presented in the middle of a computer screen with bars on the left or right edge of the screen that appeared against a black background at a viewing distance of 60 cm. Subjects were instructed to press a key when the arrow coincided in both direction and color (red, green, blue) with the bar (Go), but to withhold their response when it did not match (NoGo). The experimental design is shown in Fig. 3.

A total of 65 images from the International Affective Picture System (IAPS)^66^ were used. Thirty-five images with pleasant content and 30 others with unpleasant content were selected for the two contexts. The pleasant and unpleasant images were chosen on the basis of scores for valence and arousal using a 1-9-point analog scale from similar age groups. Images were assessed by 27 women and 17 men. The values of valence and arousal are shown in Table 2. Both the female and male subjects in the present experiment were shown the same 30 neutral, 30 unpleasant (disgust, fear, violence) and 25 pleasant (adventure, couples) images, though some of the pleasant stimuli (5) that showed opposite sex models differed for females and males. No images with explicit nudity were included. The neutral context consisted of scrambled images of the same pleasant and unpleasant images.

**Table 2:** Valence and arousal of the stimuli used in the experimental task.

	Valence			Arousal		
	PleasantM(SD)	UnpleasantM(SD)	*t-*test, df = 58	PleasantM(SD)	UnpleasantM(SD)	*t-*test, df = 58
Females	6.48 (0.74)	3.34(0.72)	t = 17.73,p < 0.001	5.23 (0.89)	4.80 (1.32)	t = 1.49,p = 0.14
Males	6.43 (0.49)	4.22 (0.55)	t = 16.54,p < 0.001	4.17 (0.71)	3.93 (0.95)	t = 1.13,p = 0.26

Stimuli duration was 300 ms and the inter-trial interval was 1500 ms. Each condition consisted of 240 trials (75% Go, 25% NoGo) and lasted approximately 6 min. The order of presentation of the conditions was randomized across subjects. The parameters measured were: percentage of correct responses, percentage of correct inhibitions, and reaction times. At the beginning of the experimental session, all participants received training to ensure that they clearly understood the task.

### Electrophysiological recording

EEG were recorded continuously at Fp1, Fp2, F3, F4, F7, F8, C3, C4, T3, T4, T5, T6, P3, P4, O1, O2, Fz, Cz and Pz, according to the 10/20 International System, with linked earlobe reference attached to an elastic cap. A Medicid 5 amplifier from Neuronic S.A. was used. The band pass was set at 0.05–50 Hz, and the sample rate at 500 Hz. Electrode impedance was kept below 5 KOhms. Electrooculograms (EOG) were recorded through electrodes placed on the supraorbital and infraorbital eye regions to eliminate segments contaminated by eye-movement artifacts. Twenty EEG artifact-free epochs from correct responses and correct inhibitions were independently averaged for each emotional condition of interest. Equal numbers of Go and NoGo trials were obtained. Epochs were defined as 100 ms before stimulus onset (baseline) and 800 ms post-stimulus. Electrode sites and scoring windows were selected by examining grand-average and individual waveforms in order to evaluate the typical recording sites analyzed in the literature for the Go/NoGo paradigm. N2 was identified as the most negative peak in the interval between 200 and 350 ms, while P3 peak amplitude was measured individually in the window between 350 and 600 ms after stimulus presentation. N2 and P3 amplitudes and latencies were analyzed for the frontal, central and parietal sites at midline (Fz, Cz and Pz) and in the left (F3, C3, P3) and right hemispheres (F4, C4, P4) because previous studies had reported higher amplitudes for N2 and P3 at these locations while performing a Go/NoGo paradigm^20, 21, 28, 29, 67^.

### Statistical analysis

A mixed factorial analysis (2 × 3) was used to evaluate the behavioral measures, including factors –sex: male, female– and context: neutral, pleasant and unpleasant. Another mixed factorial analysis was used to analyze amplitude and latency data based on the following factors: sex (male, female), trials (Go, NoGo), contexts (neutral, pleasant, unpleasant), topographic distribution (frontal, central, parietal) and laterality (left: F3, C3, P3; midline: Fz, Cz, Pz; and, right: F4, C4, P4). Greenhouse-Geisser correction was used to adjust degrees of freedom and *p*-values (p < 0.05) when appropriate, together with Bonferroni correction for pairwise comparisons.

## Electronic supplementary material

Tables 3 and 4

## References

[1] Dahl RE, Gunnar MR (2009). Heightened stress responsiveness and emotional reactivity during pubertal maturation: implications for psychopathology. Dev. Psychopathol..

[2] Casey BJ, Galvan A, Hare TA (2005). Changes in cerebral functional organization during cognitive development. Curr. Opin. Neurobiol..

[3] Steinberg L (2005). Cognitive and affective development in adolescence. Trends Cogn. Sci..

[4] Ernst M (2005). Amygdala and nucleus accumbens in responses to receipt and omission of gains in adults and adolescents. NeuroImage.

[5] Galvan A (2006). Earlier development of the accumbens relative to orbitofrontal cortex might underlie risk-taking behavior in adolescents. J. Neurosci..

[6] Spear LP (2013). Adolescent neurodevelopment. J. Adolesc. Health.

[7] Hare TA (2008). Biological substrates of emotional reactivity and regulation in adolescence during an emotional go-nogo task. Biol. Psychiatry.

[8] Jaeger A (2013). Inhibitory control and the adolescent brain: A review of fMRI research. Psy. Neuro.

[9] Rubia K (2006). Progressive increase of frontostriatal brain activation from childhood to adulthood during event-related task of cognitive control. Hum. Brain Mapp..

[10] Casey BJ, Jones RM, Hare TA (2008). The adolescent brain. Ann. N.Y. Acad. Sci.

[11] Barkley RA (1997). Behavioral inhibition, sustained attention, and executive functions: constructing a unifying theory of ADHD. Psychol. Bull..

[12] Norman AL (2011). Neural activation during inhibition predicts initiation of substance use in adolescence. Drug Alcohol Depend.

[13] Verbruggen F, De Houwer J (2007). Do emotional stimuli interfere with response inhibition? Evidence from the stop signal paradigm. Cogn. Emot.

[14] Goldstein M (2007). Neural substrates of the interaction of emotional stimulus processing and motor inhibitory control: An emotional linguistic go/no-go fMRI study. Neuroimage.

[15] Schulz KP (2007). Does the emotional go/no-go task really measure behavioral inhibition? Convergence with measures on a non-emotional analog. Arch. Clin. Neuropsychol..

[16] Shafritz KM, Collins SH, Blumberg HP (2006). The interaction of emotional and cognitive neural systems in emotionally guided response inhibition. Neuroimage.

[17] Brown MR (2012). Effects of emotional context on impulse control. Neuroimage.

[18] Brown MR (2015). Neural correlates of high-risk behavior tendencies and impulsivity in an emotional Go/NoGo fMRI task. Front. Syst. Neurosci.

[19] Aron AR, Robbins TW, Poldrack RA (2004). Inhibition and the right frontal cortex. Trends Cogn. Sci..

[20] Bokura H, Yamaguchi S, Kobayashi S (2001). Electrophysiological correlates for response inhibition in a Go/NoGo task. Clin. Neurophysiol..

[21] Folstein JR, Van Petten C (2008). Influence of cognitive control and mismatch on the N2 component of the ERP: A review. Psychophysiology.

[22] Zhang W, Lu J (2012). Time course of automatic emotion regulation during a facial Go/Nogo task. Biol. Psychol..

[23] Bruin KJ, Wijers AA (2002). Inhibition, response mode, and stimulus probability: a comparative event-related potential study. Clin. Neurophysiol.

[24] Nieuwenhuis S, Yeung N, van den Wildenberg W, Riddenrinkhof KR (2003). Electrophysiological correlates of anterior cingulate function in a go/no-go task: Effects of response conflict and trial type frequency. Cogn. Affect Behav. Neurosci..

[25] Benikos N, Johnstone SJ, Roodenrys SJ (2013). Varying task difficulty in the Go/Nogo task: the effects of inhibitory control, arousal, and perceived effort on ERP components. Int. J. Psychophysiol..

[26] Géczy I, Czigler I (1999). & Balázs. Effects of cue information on response production and inhibition measured by event-related potentials. Acta Physiol. Hung.

[27] Donchin E, Coles MG (1988). Is the P300 component a manifestation of context updating?. Behav. Brain Sci..

[28] Barry RJ, Rushby JA (2006). An orienting reflex perspective on anteriorisation of the P3 of the event-related potential. Exp. Brain Res..

[29] Polich J (2007). Updating P300: An Integrative Theory of P3a and P3b. Clin. Neurophysiol..

[30] Bruin KJ, Wijers AA, van Staveren AS (2001). Response priming in a go/nogo task: do we have to explain the go/nogo N2 effect in terms of response activation instead of inhibition?. Clin. Neurophysiol..

[31] Gajewski PD, Falkenstein M (2013). Effects of task complexity on ERP components in Go/NoGo tasks. Int. J. Psychophysiol..

[32] Smith JL, Johnstone SJ, Barry RJ (2008). Movement-related potentials in the Go/NoGo task: the P3 reflects both cognitive and motor inhibition. Clin. Neurophysiol..

[33] Kok A, Ramautar JR, de Ruiter MB, Band GP, Ridderinkhof KR (2004). ERP components associated with successful and unsuccessful stopping in a stop-signal task. Psychophysiology.

[34] Verleger R, Paehge T, Kolev V, Yordanova J, Jaœkowski P (2009). On the relation of movement-related potentials to the go/no-go effect on P3. Biol. Psychol..

[35] Dimoska A, Johnstone SJ, Barry RJ, Clarke AR (2003). Inhibitory motor control in children with attention-deficit/hyperactivity disorder: event-related potentials in the stop-signal paradigm. Biol. Psychiatry.

[36] Overtoom CCE (2002). Inhibition in children with attention-deficit/hyperactivity disorder: a psychophysiological study of the stop task. Biol. Psychiatry.

[37] Huster RJ (2013). Electroencephalography of response inhibition tasks: functional networks and cognitive contributions. Int. J. Psychophysiol..

[38] Albert J, López-Martín S, Carretié L (2010). Emotional context modulates response inhibition: neural and behavioral data. Neuroimage.

[39] Albert J (2012). The role of the anterior cingulate cortex in emotional response inhibition. Hum. Brain Mapp..

[40] Buodo G (2017). Unpleasant stimuli differentially modulate inhibitory processes in an emotional Go/NoGo task: an event related potential study. Cogn. Emot.

[41] Killgore WD, Oki M, Yurgelun-Todd DA (2001). Sex-specific developmental changes in amygdala responses to affective faces. Neuroreport.

[42] Xu M (2015). The divergent effects of fear and disgust on unconscious inhibitory control. Cogn. Emot.

[43] Shulman EP, Harden KP, Chein JM, Steinberg L (2015). Sex differences in the developmental trajectories of impulse control and sensation-seeking from early adolescence to early adulthood. J. Youth Adolesc.

[44] Lenroot RK (2007). Sexual dimorphism of brain developmental trajectories during childhood and adolescence. Neuroimage.

[45] Bianchin M, Angrilli A (2011). Decision preceding negativity in the Iowa Gambling Task: an ERP study. Brain Cogn..

[46] Bradley MM, Codispoti M, Sebatinelli D, Lang PJ (2001). Emotion and motivation II: sex differences in picture processing. Emotion.

[47] Lithari C (2010). Are females more responsive to emotional stimuli? A neurophysiological study across arousal and valence. Brain Topogr..

[48] Gardener EK, Carr AR, Macgregor A, Felmingham KL (2013). Sex differences and emotion regulation: an event-related potential study. PLoS One.

[49] Cohen-Gilbert JE, Thomas KM (2013). Inhibitory control during emotional distraction across adolescence and early adulthood. Child Dev..

[50] Singhal A (2012). Electrophysiological correlates of fearful and sad distraction on target processing in adolescents with attention deficit-hyperactivity symptoms and affective disorders. Front. Integr. Neurosci.

[51] Ramos-Loyo J, Angulo-Chavira A, Llamas-Alonso LA, González-Garrido AA (2016). Sex differences in emotional contexts modulation on response inhibition. Neuropsychologia.

[52] Yurgelun-Todd D (2007). Emotional and cognitive changes during adolescence. Curr. Opin. Neurobiol..

[53] Dempster FN (1992). The rise and fall of the inhibitory mechanism: Toward a unified theory of cognitive development and aging. Dev. Rev..

[54] Hare TA (2005). Contributions of amygdala and striatal activity in emotion regulation. Biol. Psychiatry.

[55] Chiu PH, Holmes AJ, Pizzagalli DA (2008). Dissociable recruitment of rostral anterior cingulate and inferior frontal cortex in emotional response inhibition. Neuroimage.

[56] Pfefferbaum A, Ford JM, Weller BJ, Kopell BS (1985). ERPs to response production and inhibition. Electroencephalogr. Clin. Neurophysiol..

[57] Fallgater AJ, Strik WK (1999). The NoGo-anteriorization as a neurophysiological standard-index for cognitive response control. Int. J. Psychophysiol..

[58] Schmajuk M, Liotti M, Busse L, Woldorff MG (2006). Electrophysiological activity underlying inhibitory control processes in normal adults. Neuropsychologia.

[59] Smith JL, Johnstone SJ, Barry RJ (2007). Response priming in the Go/NoGo task: the N2 reflects neither inhibition nor conflict. Clin. Neurophysiol..

[60] Salisbury DF, Griggs CB, Shenton ME, McCarley RW (2004). The NoGo P300 anteriorization effect and response inhibition. Clin. Neurophysiol..

[61] Delplanque S, N’diaye K, Scherer K, Grandjean D (2007). Spatial frequencies or emotional effects? A systematic measure of spatial frequencies for IAPS pictures by a discrete wavelet analysis. J. Neurosci. Methods.

[62] Hartikainen KM, Ogawa KH, Knight RT (2000). Transient interference or right hemispheric function due to automatic emotional processing. Neuropsychologia.

[63] Tipples J, Sharma D (2000). Orienting to exogenous cues and attentional bias to affective pictures reflect separate processes. Br. J. Psychol.

[64] Ohman A, Flykt A, Esteves F (2001). Emotion drives attention: detecting the snake in the grass. J. Exp. Psychol. Gen.

[65] Li CS, Huang C, Constable RT, Sinha R (2006). Gender differences in the neural correlates of response inhibition during a stop signal task. Neuroimage.

[66] Lang, P. J., Bradley, M. M. & Cuthbert, B. N. International affective picture system (IAPS): Technical manual and affective ratings University of Florida, Center for Research in Psychophysiology, Gainesville (1999).

[67] Cuthbert BN, Schupp HT, Bradley MM, Birbaumer N, Lang PJ (2000). Brain potentials in affective picture processing: covaration with autonomic arousal and affective report. Biol. Psychol..

