# Supplementary material for: Emotional Contexts Exert a Distracting Effect on Attention and Inhibitory Control in Female and Male Adolescents
Source: Sci Rep. 2017 May 18;7:2082. doi: 10.1038/s41598-017-02020-8 (PMC5437040; doi:10.1038/s41598-017-02020-8)
Supplement: Supplementary file 1 — Tables 3 and 4 [file 41598_2017_2020_MOESM1_ESM.doc]

**Emotional Contexts Exert a Distracting Effect on Attention and Inhibitory Control in Female and Male Adolescents**

Julieta Ramos-Loyo^1^,*, Luis A. Llamas-Alonso^1^, Andrés A. González-Garrido^1^, Juan Hernández-Villalobos^1^

^1^Instituto de Neurociencias, Universidad de Guadalajara, Guadalajara, Jalisco, México

Table 3. N2 and P3 amplitude values (means and standard deviations) in each condition: neutral (NC), pleasant (PC) and unpleasant (UC) for females and males.

| **N2Go Amplitude** | | | | | | | | | |
| --- | --- | --- | --- | --- | --- | --- | --- | --- | --- |
|  | **Average** | | | **Females** | | | **Males** | | |
|  | **NC** | **PC** | **UC** | **NC** | **PC** | **UC** | **NC** | **PC** | **UC** |
| **F3** | -5.6(3.6) | -6.5(3.2) | -8.4(4.3) | -5(4) | -6.4(3.1) | -8.9(4.6) | -6.3(3.2) | -6.5(3.3) | -8.0(4.1) |
| **F4** | -6.6(3.6) | -7.3(3.8) | -9.3(5.1) | -5.9(3.4) | -6.8(4) | -9.4(4.2) | -7.2(3.8) | -7.8(3.7) | -9.2(6) |
| **FZ** | -7.0(3.7) | -8.2(3.6) | -9.6(4.6) | -6.6(3.9) | -8.2(3.6) | -10.2(4.4) | -7.5(3.5) | -8.2(3.7) | -9.1(4.9) |
| **C3** | -5.2(3.1) | -5.6(3) | -7.8(4.5) | -4.4(3.1) | -5.5(3.4) | -7.8(4.9) | -6.1(3) | -5.7(2.7) | -7.9(4.2) |
| **C4** | -5.6(2.8) | -6.2(3.1) | -8.1(4.1) | -5.2(3) | -5.7(3.4) | -8.2(3.7) | -6.1(2.7) | -6.7(2.7) | -8.1(4.6) |
| **CZ** | -7.0(3.6) | -7.9(3.5) | -9.7(4.6) | -6.7(3.9) | -8.1(3.5) | -10.1(4) | -7.4(3.5) | -7.7(3.5) | -9.4(5.2) |
| **P3** | -2.9(3.4) | -2.4(3.9) | -3.6(4.7) | -2(3.1) | -1.9(3.5) | -3.1(4.7) | -3.7(3.6) | -2.9(4.3) | -4.1(4.9) |
| **P4** | -3.3(2.9) | -3.2(3.5) | -3.9(3.4) | -2.7(3.4) | -2.1(3.3) | -3.6(3.4) | -3.8(2.3) | -4.2(3.4) | -4.2(3.6) |
| **PZ** | -4.4(3.4) | -4.4(3) | -5.9(3.7) | 3.6(3) | -3.8(2.9) | -5.3(3.4) | -5.2(3.9) | -5.0(3.1) | -6.6(4.1) |
| **N2NoGo Amplitude** | | | | | | | | | |
|  | **Average** | | | **Females** | | | **Males** | | |
|  | **NC** | **PC** | **UC** | **NC** | **PC** | **UC** | **NC** | **PC** | **UC** |
| **F3** | -5.3(4.3) | -6.4(3.3) | -7.2(5.3) | -4.8(3.7) | -6.0(3.8) | -7.4(4.7) | -5.8(4.8) | -6.8(2.8) | -7.1(5.9) |
| **F4** | -6.1(4.3) | -7.1(3.4) | -8.9(4.5) | -5.4(3.1) | -6.8(3.3) | -8.1(4.5) | -6.6(5.2) | -7.2(3.6) | -9.7(4.5) |
| **FZ** | -7.6(4.9) | -8.7(3.9) | -10.6(4.5) | -8.9(4.9) | -9.7(4.4) | -11.6(4.8) | -6.2(4.6) | -7.7(3.2) | -9.6(3.9) |
| **C3** | -5.9(3.7) | -7.2(4.3) | -9.0(4.7) | -5.9(3.6) | -7.4(5.5) | -8.9(5.7) | -5.9(3.9) | -7.1(2.7) | -9.1(3.7) |
| **C4** | -6.2(3.8) | -7.2(4.1) | -9.2(4.9) | -6.2(3.8) | -7.6(5.4) | -9.5(5.5) | -6.1(3.9) | -6.9(2.4) | -8.8(4.3) |
| **CZ** | -8.1(4.4) | -8.9(3.5) | -11.1(3.9) | -8.9(4.2) | -9.6(3.3) | -11.7(3.5) | -7.2(4.5) | -8.3(3.6) | -10.3(4.3) |
| **P3** | -2.9(3.2) | -3.7(3.8) | -3.9(3.9) | -2.5(3.2) | -2.4(4.1) | -2.5(3.6) | -3.3(3.3) | -4.9(3) | -5.2(3.8) |
| **P4** | -2.9(2.5) | -3.6(3.6) | -4.1(4) | -2.9(2.6) | -3.0(3.9) | -3.5(3.7) | -2.9(2.5) | -4.2(3.1) | -4.7(4.3) |
| **PZ** | -4.6(3.4) | -5.5(2.9) | -6.1(3.6) | -4.8(3.2) | -5.0(2.9) | -5.6(3) | -4.5(3.7) | -5.9(2.9) | -6.6(4.1) |
| **P3Go Amplitude** | | | | | | | | | |
|  | **Average** | | | **Females** | | | **Males** | | |
|  | **NC** | **PC** | **UC** | **NC** | **PC** | **UC** | **NC** | **PC** | **UC** |
| **F3** | 5.1(3.2) | 5.4(3.6) | 4.1(2.9) | 4.6(3.9) | 5.2(3.8) | 4.1(2.7) | 5.6(2.3) | 5.6(3.5) | 4.1(3) |
| **F4** | 5(3.4) | 5.3(3.4) | 3.9(3.6) | 4.0(3.6) | 5.8(4.1) | 3.8(2.4) | 6.1(2.9) | 4.9(2.6) | 4.1(4.6) |
| **FZ** | 4.5(3.5) | 4.6(3.6) | 3.3(3.4) | 3.3(3) | 4.3(3.8) | 2.2(2.1) | 5.7(2.4) | 4.8(3.4) | 4.3(3.8) |
| **C3** | 6.4(3.5) | 7.0(3.8) | 6.1(2.6) | 6.3(4) | 7.9(3.5) | 6.1(2.5) | 6.4(3) | 6.2(4.1) | 6.3(2.7) |
| **C4** | 6.3(3.6) | 7.7(3.9) | 6.4(2.3) | 5.3(2.8) | 8.3(3.1) | 6.2(2.5) | 7.3(4.1) | 6.8(4.5) | 6.6(2.2) |
| **CZ** | 6.8(4) | 7.2(3.8) | 6.7(3.2) | 5.7(4) | 7.3(3.2) | 6.1(3.2) | 7.9(3.7) | 7.1(4.5) | 7.4(3.2) |
| **P3** | 7.1(2.9) | 6.7(4) | 6.3(2.9) | 6.9(3.4) | 7.4(3.6) | 6.7(2.9) | 7.4(2.4) | 6.0(4.3) | 6.0(3.1) |
| **P4** | 6.3(3) | 6.5(3.8) | 5.9(2.5) | 5.5(2.5) | 6.9(3) | 5.8(2.5) | 7.0(3.4) | 6.0(4.4) | 6.0(2.6) |
| **PZ** | 7.3(3.5) | 7.4(3.9) | 7.0(2.3) | 6.9(3.9) | 8.2(3) | 7.2(2.4) | 7.6(3.1) | 6.5(4.5) | 6.8(2.3) |
| **NoGo Amplitude P3** | | | | | | | | | |
|  | **Average** | | | **Females** | | | **Males** | | |
|  | **NC** | **PC** | **UC** | **NC** | **PC** | **UC** | **NC** | **PC** | **UC** |
| **F3** | 9.7(4.3) | 9.8(4.5) | 9.0(4) | 11(4) | 11.0(4.3) | 10.3(3.8) | 8.5(4.3) | 8.6(4.4) | 7.6(3.9) |
| **F4** | 10.2(4.8) | 10.1(4.4) | 9.1(4.1) | 10.8(5.1) | 10.8(4.4) | 10.4(3.7) | 9.6(4.5) | 9.3(4.3) | 7.7(4.1) |
| **FZ** | 9.6(5) | 9.1(4.1) | 8.3(4.1) | 9.1(3.7) | 8.6(4.2) | 8.1(4.3) | 10.2(4.5) | 9.5(4.1) | 8.5(3.9) |
| **C3** | 9.5(4.5) | 9.6(5) | 8.7(3.8) | 8.4(3.9) | 7.8(4.8) | 7.6(3.5) | 10.7(4.9) | 11.5(4.7) | 9.7(4.0) |
| **C4** | 10.5(5.7) | 10.8(5.5) | 9.5(4.4) | 8(4.5) | 8.4(4.5) | 7.2(3.7) | 12.9(5.9) | 13.2(5.6) | 11.8(4.0) |
| **CZ** | 14.0(5.7) | 14.4(5.4) | 13.1(4.9) | 14(6) | 14.3(5.4) | 12.7(5.2) | 14.1(5.5) | 14.5(5.5) | 13.6(4.7) |
| **P3** | 8.8(3.6) | 8.9(3.6) | 8.4(3.3) | 9.6(3.8) | 9.5(3.3) | 9.2(2.9) | 8.0(3.4) | 8.4(3.8) | 7.6(3.6) |
| **P4** | 9.4(4.9) | 9.2(4.4) | 9.0(3.5) | 9.3(4.7) | 9.4(3.6) | 9.1(3.5) | 9.5(5.1) | 9.0(5.2) | 8.8(3.6) |
| **PZ** | 10.8(4.3) | 10.9(5.4) | 9.5(5.2) | 10.9(4) | 10.8(4) | 10.3(3.8) | 10.8(4.5) | 11(5.4) | 8.7(6.1) |

Table 4. N2 and P3 latency values (means and standard deviations) in each condition: neutral (NC), pleasant (PC) and unpleasant (UC) for females and males.

| **N2Go Latency** | | | | | | | | | |
| --- | --- | --- | --- | --- | --- | --- | --- | --- | --- |
|  | **Average** | | | **Females** | | | **Males** | | |
|  | **NC** | **PC** | **UC** | **NC** | **PC** | **UC** | **NC** | **PC** | **UC** |
| **F3** | 245(53) | 255(33) | 258(37) | 249(66) | 265(31) | 261(46) | 240(37) | 246(33) | 255(28) |
| **F4** | 243(47) | 255(37) | 259(34) | 253(53) | 265(36) | 267(40) | 233(38) | 265(36) | 252(26) |
| **FZ** | 242(40) | 261(35) | 265(29) | 249(44) | 278(23) | 277(26) | 234(35) | 244(37) | 253(28) |
| **C3** | 250(45) | 263(32) | 259(34) | 257(52) | 267(34) | 265(44) | 243(39) | 259(30) | 252(20) |
| **C4** | 241(42) | 255(34) | 261(34) | 251(49) | 269(29) | 269(40) | 231(32) | 242(35) | 253(25) |
| **CZ** | 245(40) | 266(32) | 267(27) | 255(46) | 278(27) | 281(25) | 236(31) | 253(34) | 252(22) |
| **P3** | 227(46) | 252(39) | 253(40) | 233(56) | 266(34) | 270(41) | 221(33) | 238(40) | 236(33) |
| **P4** | 234(45) | 243(41) | 254(42) | 242(52) | 264(29) | 271(34) | 226(37) | 222(41) | 236(43) |
| **PZ** | 246(44) | 256(34) | 255(54) | 257(53) | 273(24) | 280(27) | 234(29) | 240(36) | 230(63) |
| **N2NoGo Latency** | | | | | | | | | |
|  | **Average** | | | **Females** | | | **Males** | | |
|  | **NC** | **PC** | **UC** | **NC** | **PC** | **UC** | **NC** | **PC** | **UC** |
| **F3** | 250(42) | 274(49) | 262(33) | 256(45) | 290(42) | 279(25) | 243(38) | 258(52) | 246(32) |
| **F4** | 249(46) | 283(44) | 264(36) | 257(47) | 294(44) | 280(23) | 241(44) | 271(43) | 247(39) |
| **FZ** | 258(49) | 277(34) | 263(29) | 272(53) | 282(23) | 275(19) | 243(40) | 272(42) | 251(33) |
| **C3** | 234(42) | 269(35) | 262(29) | 236(48) | 268(30) | 274(21) | 232(38) | 269(41) | 250(31) |
| **C4** | 246(44) | 272(40) | 257(35) | 250(51) | 277(36) | 268(33) | 243(37) | 267(44) | 245(35) |
| **CZ** | 263(46) | 279(34) | 269(25) | 279(49) | 289(23) | 280(23) | 248(38) | 269(41) | 258(23) |
| **P3** | 242(49) | 264(51) | 260(34) | 258(45) | 276(53) | 273(31) | 226(48) | 251(48) | 247(32) |
| **P4** | 252(47) | 275(47) | 268(33) | 264(49) | 286(48) | 282(23) | 240(43) | 265(45) | 253(35) |
| **PZ** | 260(51) | 275(42) | 274(38) | 277(50) | 291(40) | 293(37) | 243(48) | 259(39) | 254(28) |
| **P3Go Latency** | | | | | | | | | |
|  | **Average** | | | **Females** | | | **Males** | | |
|  | **NC** | **PC** | **UC** | **NC** | **PC** | **UC** | **NC** | **PC** | **UC** |
| **F3** | 472(84) | 517(91) | 485(80) | 483(84) | 517(89) | 515(78) | 462(86) | 517(96) | 454(72) |
| **F4** | 464(79) | 521(85) | 494(76) | 467(73) | 541(83) | 516(76) | 462(85) | 502(84) | 473(72) |
| **FZ** | 461(75) | 528(76) | 500(82) | 456(68) | 539(72) | 534(72) | 465(83) | 517(80) | 465(79) |
| **C3** | 460(78) | 517(67) | 483(69) | 460(80) | 535(37) | 496(67) | 459(79) | 500(85) | 470(70) |
| **C4** | 453(69) | 520(61) | 488(68) | 464(68) | 540(44) | 498(73) | 441(70) | 500(70) | 478(63) |
| **CZ** | 471(62) | 530(72) | 504(71) | 473(55) | 546(64) | 540(50) | 469(70) | 515(79) | 468(73) |
| **P3** | 420(70) | 473(79) | 453(67) | 424(83) | 491(71) | 468(66) | 415(57) | 455(85) | 438(67) |
| **P4** | 416(71) | 471(72) | 458(72) | 423(84) | 493(59) | 474(76) | 409(58) | 448(79) | 441(66) |
| **PZ** | 423(62) | 484(78) | 464(71) | 431(71) | 508(82) | 478(74) | 414(52) | 460(67) | 450(68) |
| **P3NoGo Latency** | | | | | | | | | |
|  | **Average** | | | **Females** | | | **Males** | | |
|  | **NC** | **PC** | **UC** | **NC** | **PC** | **UC** | **NC** | **PC** | **UC** |
| **F3** | 533(55) | 554(44) | 561(53) | 520(69) | 558(53) | 560(46) | 547(30) | 550(35) | 563(60) |
| **F4** | 542(56) | 561(47) | 573(51) | 535(72) | 572(42) | 586(47) | 549(34) | 550(50) | 560(52) |
| **FZ** | 546(32) | 549(43) | 561(47) | 543(35) | 549(35) | 550(49) | 548(28) | 550(48) | 572(43) |
| **C3** | 544(47) | 558(46) | 580(56) | 545(62) | 559(56) | 588(55) | 543(28) | 556(35) | 571(40) |
| **C4** | 540(59) | 567(46) | 579(59) | 537(75) | 572(55) | 589(69) | 543(39) | 562(35) | 570(45) |
| **CZ** | 540(36) | 555(33) | 563(44) | 541(35) | 551(32) | 556(49) | 539(39) | 558(35) | 570(39) |
| **P3** | 512(76) | 528(67) | 546(70) | 499(87) | 507(79) | 542(60) | 525(62) | 549(46) | 550(80) |
| **P4** | 520(80) | 541(75) | 541(76) | 499(100) | 522(91) | 532(80) | 540(48) | 561(52) | 550(73) |
| **PZ** | 539(47) | 555(32) | 560(66) | 538(50) | 553(30) | 561(43) | 540(46) | 556(35) | 558(84) |
